# Respiratory frequency and tidal volume during exercise: differential control and unbalanced interdependence

**DOI:** 10.14814/phy2.13908

**Published:** 2018-11-04

**Authors:** Andrea Nicolò, Michele Girardi, Ilenia Bazzucchi, Francesco Felici, Massimo Sacchetti

**Affiliations:** ^1^ Department of Movement, Human and Health Sciences University of Rome “Foro Italico” Rome Italy

**Keywords:** Breathing control, exercise hyperpnoea, perceived exertion, sinusoidal exercise, ventilatory pattern

## Abstract

Differentiating between respiratory frequency (*f*_R_) and tidal volume (*V*_T_) may improve our understanding of exercise hyperpnoea because *f*_R_ and *V*_T_ seem to be regulated by different inputs. We designed a series of exercise manipulations to improve our understanding of how *f*_R_ and *V*_T_ are regulated during exercise. Twelve cyclists performed an incremental test and three randomized experimental sessions in separate visits. In two of the three experimental visits, participants performed a moderate‐intensity sinusoidal test followed, after recovery, by a moderate‐to‐severe‐intensity sinusoidal test. These two visits differed in the period of the sinusoid (2 min vs. 8 min). In the third experimental visit, participants performed a trapezoidal test where the workload was self‐paced in order to match a predefined trapezoidal template of rating of perceived exertion (RPE). The results collectively reveal that *f*_R_ changes more with RPE than with workload, gas exchange, *V*_T_ or the amount of muscle activation. However, *f*_R_ dissociates from RPE during moderate exercise. Both *V*_T_ and minute ventilation (${\dot{V}}_{E}$) showed a similar time course and a large correlation with $\dot{V}{\text{CO}}_{2}$in all the tests. Nevertheless, $\dot{V}{\text{CO}}_{2}$ was associated more with ${\dot{V}}_{E}$ than with *V*_T_ because *V*_T_ seems to adjust continuously on the basis of *f*_R_ levels to match ${\dot{V}}_{E}$ with $\dot{V}{\text{CO}}_{2}$. The present findings provide novel insight into the differential control of *f*_R_ and *V*_T_ – and their unbalanced interdependence – during exercise. The emerging conceptual framework is expected to guide future research on the mechanisms underlying the long‐debated issue of exercise hyperpnoea.

## Introduction

Understanding how ventilation is regulated during exercise is a classical challenge in respiratory physiology. Emerging evidence suggests that differentiating between the two components of minute ventilation can help shed some light on the control of breathing because the different inputs regulating ventilation seem to act separately on respiratory frequency (*f*_R_) and tidal volume (*V*_T_) (Nicolò et al. 2017b,a; Tipton et al. 2017). It has been proposed that, during exercise, *f*_R_ is mainly regulated by fast inputs (including central command) (Nicolò et al. 2017b), and this may help explain why *f*_R_ and perceived exertion are closely associated during a variety of exercise conditions (Robertson and Noble 1997; Nicolò et al. 2014, 2016, 2017b). Conversely, *V*_T_ appears to be mainly regulated by metabolic stimuli (Nicolò et al. 2017a,b). However, very little research has been conducted with the purpose of investigating the differential control of *f*_R_ and *V*_T_ during exercise, making our current understanding of this issue limited.

Our partial understanding of exercise hyperpnoea is also due to the fact that the putative inputs driving ventilation cannot be experimentally isolated during “real” exercise conditions (Forster et al. 2012). A suitable solution which partly addresses this problem is the manipulation of exercise protocols as experimental interventions (Casaburi et al. 1977; Nicolò et al. 2017b). A classical protocol used to study the control of ventilation is the sinusoidal exercise, where relevant physiological responses can be conveniently analyzed in terms of amplitude and phase lag from the input (workload), in order to discern and rigorously quantitate relationships between the perturbing workload and the responding variables (Casaburi et al. 1977; Haouzi 2006). The sinusoidal exercise shows a close association between minute ventilation (${\dot{V}}_{E}$) and $\dot{V}{\text{CO}}_{2}$ during moderate intensity, with both variables showing a substantial phase lag compared to variations in workload (Casaburi et al., 1977, 1995; Bakker et al. 1980; Miyamoto et al. 1983; Haouzi 2006; Fukuoka et al. 2017). This is considered strong evidence in favor of ventilation following the changes in metabolism while ignoring muscle afferent feedback and central command inputs (Haouzi, 2006, 2015; Forster et al. 2012), with the possible exception of muscle afferent feedback sensing vascular distension (Haouzi 2006). While this may hold true during moderate intensity, it is unclear how ventilation responds to sinusoidal changes in workload during high‐intensity exercise, where the magnitude of central command is higher. Furthermore, in the light of the proposition that *f*_R_ is not substantially regulated by metabolic stimuli (Nicolò et al. 2017a,b), it is conceivable that *V*_T_ mediates the close association observed between ${\dot{V}}_{E}$ and $\dot{V}{\text{CO}}_{2}$. However, there are only a few underappreciated reports on how *f*_R_ and *V*_T_ respond to sinusoidal changes in workload (Bakker et al. 1980; Miyamoto et al. 1983). Therefore, further research is needed to exploit the potential of the sinusoidal protocol and to improve our understanding of exercise hyperpnoea across different exercise‐intensity domains.

Further insight into how *f*_R_ is regulated during exercise is expected to come from elucidating the mechanisms underlying the close association between *f*_R_ and perceived exertion (Nicolò et al., 2016, 2017b). Although generally overlooked in respiratory physiology, there is a convenient exercise paradigm which allows for experimental manipulation of perceived exertion by asking the participants to self‐pace the workload in order to exercise at predetermined levels of rating of perceived exertion (RPE). This exercise modality is known as RPE production mode (Robertson and Noble 1997), and it has often been used at fixed levels of RPE. When RPE is fixed at relatively high levels, power output decreases over time together with several physiological variables including $\dot{V}O_{2}$, ${\dot{V}}_{E}$ and heart rate (HR), while *f*_R_ remains relatively stable (Cochrane et al. 2015). More convincing evidence of the existence of a mechanistic link between *f*_R_ and perceived exertion would come from verifying whether *f*_R_ follows structured variations in RPE within a single exercise test. Indeed, a variable‐RPE production‐mode test would decrease the chance of finding a spurious association between RPE and *f*_R_, and would favor the identification of those variables which are not associated with RPE. For instance, this exercise paradigm may clarify to what extent variations in *f*_R_ and RPE are associated with changes in the neuromuscular activity levels.

The present study aims to further our understanding of the control of *f*_R_ and *V*_T_ during exercise by evaluating the effect of exercise intensity, sinusoidal exercise periods, and perceived exertion levels on *f*_R_ and *V*_T_. To this end, participants performed sinusoidal exercise encompassing different exercise‐intensity domains and a novel trapezoidal exercise paradigm where the workload was self‐paced in order to match a trapezoidal template of RPE. Cardiorespiratory, mechanical, perceptual, electromyographic (EMG), and gas exchange variables were measured throughout the experimental tests. The present design was intended to test the following hypotheses: (1) *f*_R_ is closely associated with perceived exertion irrespective of the exercise test, but not with workload, metabolic markers or the amount of activation of exercising muscles; (2) *V*_T_ mediates the strong association observed between ${\dot{V}}_{E}$ and $\dot{V}{\text{CO}}_{2}$. These findings were expected to provide a novel framework for understanding how *f*_R_ and *V*_T_ are regulated during exercise.

## Methods

### Ethical approval

This study was approved by the Ethics Committee of the University of Rome Sapienza in compliance with the *Declaration of Helsinki*. Written informed consent was obtained from all participants.

### Participants

Twelve male cyclists (mean ± SD: age 22.9 ± 2.5 years, stature 177.5 ± 6.5 cm, body mass 68.6 ± 7.1 kg) volunteered to participate in this study. All the participants were well‐trained competitive cyclists. They were asked to refrain from strenuous exercise, consumption of alcohol and caffeine for at least 24 h before each test.

### Experimental overview

Participants reported to the laboratory on 4 or 5 separate occasions over a three‐week period, with visits separated by at least 48 h. On the first visit, participants performed a preliminary ramp incremental test to obtain the peak power output (PPO) and the first ventilatory threshold (VT1). After recovering from the incremental test, participants were familiarized with the experimental protocols and procedures of the following visits. Eight of 12 participants required an extra familiarization visit, as described below. Three experimental visits were then performed in random order. In two of the three experimental visits, participants performed two sinusoidal tests with different workloads on each visit, separated by 30 min of recovery. The first of the two sinusoidal tests was performed at moderate intensity while the second test was performed at moderate‐to‐severe intensity. The two sinusoidal experimental visits differed in the period of the sinusoid (2 min vs. 8 min). In the third experimental visit, participants performed a trapezoidal test where the workload was self‐paced by the participant in order to match a predefined trapezoidal template of RPE. All the tests were performed on an electromagnetically braked cycle ergometer (Lode Excalibur Sport, Groningen, the Netherlands). For each participant, the positions of the ergometer were adjusted and recorded during the first visit and were reproduced during subsequent visits. Cardiorespiratory, mechanical, perceptual, EMG, and gas exchange variables were recorded continuously during all tests.

### Preliminary ramp incremental test and familiarization trials

Before the ramp incremental test, participants were given standard instructions for providing RPE using the Borg 6–20 scale (Borg 1998). At the beginning of the ramp incremental test, participants were asked to rate their perceived exertion as soon as they were ready. Thereafter, participants were instructed to provide an RPE value by indicating a point on the RPE scale (conveniently situated close to the handlebars) whenever they felt a change in perceived exertion. This is a convenient approach for obtaining detailed information on RPE while avoiding the potential drawback of asking for RPE at too many fixed time points (de Morree and Marcora 2012). One researcher was given the sole task of recording both the reported RPE value and the corresponding moment in time. The same approach was used to measure RPE during the sinusoidal tests.

The ramp incremental test to exhaustion was preceded by a 5 min warm‐up at 100 W, 3 min of rest, and 2 min pedaling at 20 W. Subsequently, the workload increased by 30 W min^−1^. Preferred pedaling cadence was selected by each participant and was kept relatively constant for the entire test, which terminated when cadence fell by more than 10 rpm, despite strong verbal encouragement. The PPO was defined as the highest power output achieved at exhaustion, and the $\dot{V}O_{2}$_peak_ as the highest value of a 30‐sec moving average. As previously reported (Nicolò et al., 2014, 2016), the workload corresponding to VT1 was obtained from a cluster of measures including (1) the first disproportionate increase in carbon dioxide output ($\dot{V}{\text{CO}}_{2}$) from a visual inspection of individual plots of $\dot{V}{\text{CO}}_{2}$ versus $\dot{V}O_{2}$, (2) an increase in ${\dot{V}}_{E}$/$\dot{V}O_{2}$ with no increase in ${\dot{V}}_{E}$/$\dot{V}{\text{CO}}_{2}$ and (3) an increase in end‐tidal PO_2_ (P_ETO2_) with no fall in end‐tidal PCO_2_ (P_ETCO2_). The mean response time of $\dot{V}O_{2}$ was assumed to approximate 40 sec.

After having recovered from the ramp incremental test, participants were familiarized with the procedures and tests of the experimental visits. Specifically, participants performed the first portion of all the sinusoidal tests and the first trapezoidal bout of the trapezoidal test as a preliminary familiarization. To guarantee a correct execution of the trapezoidal experimental test, 8 of 12 participants were asked to perform an extra familiarization visit where they performed the entire trapezoidal test.

### Sinusoidal tests

**Figure 1:**
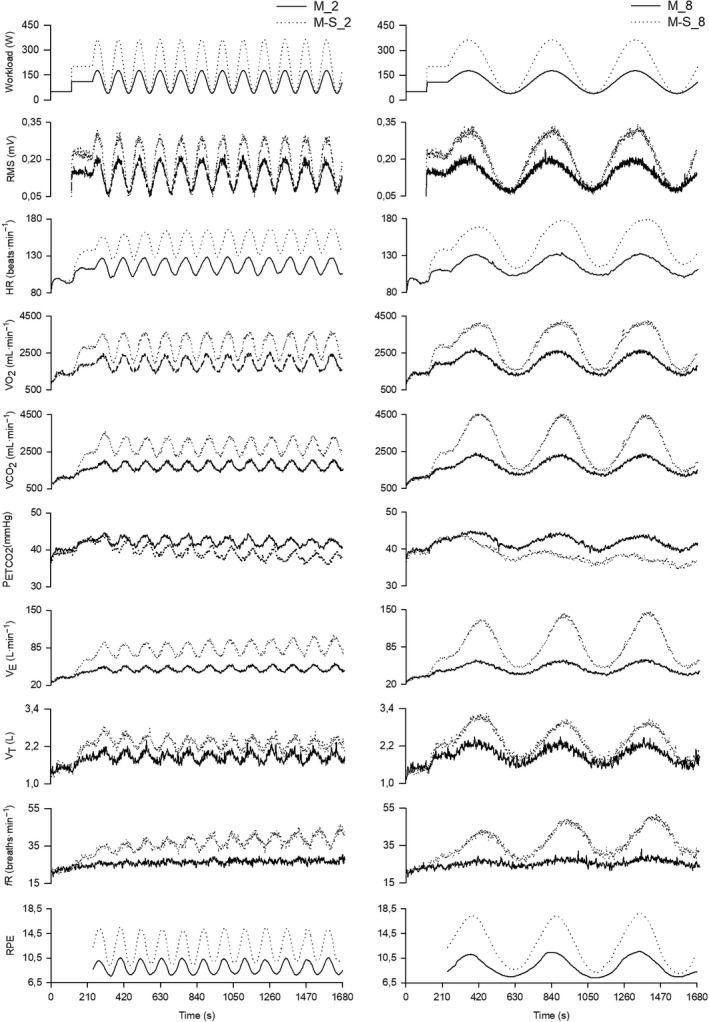
Group mean response of mechanical, physiological and perceptual variables during the four sinusoidal tests. Left panels show variables during M_2 (solid line) and M‐S_2 (dotted line), while right panels show variables during M_8 (solid line) and M‐S_8 (dotted line). The Figure depicts filtered second‐by‐second data.

Four sinusoidal tests were performed in two separate experimental visits. During each experimental visit, participants performed a sinusoidal test at moderate intensity followed by a sinusoidal test at moderate‐to‐severe intensity, separated by 30 min of recovery. The two sinusoidal experimental visits were performed with the same workload but differed in the sinusoidal period (2 min vs. 8 min). Each sinusoidal test lasted 28 min (2 min at 20 W, 2 min at a constant workload corresponding to the midpoint between the zenith and nadir of the sinusoidal variations, and 24 min of sinusoidal fluctuations in workload). Therefore, 12 and 3 complete sinusoidal cycles were performed for the 2‐min period tests and the 8‐min period tests, respectively. During the sinusoidal fluctuations, workload varied from 40 W to 90% of VT1 for the moderate‐intensity tests (M_2 and M_8) and from 40 W to 70% Δ (i.e., VT1 + 70% of the difference between PPO and VT1) for the moderate‐to‐severe‐intensity tests (M‐S_2 and M‐S_8). 90% of VT1 and 70% Δ are situated in the moderate‐ and severe‐intensity domains, respectively (Lansley et al. 2011). For each participant, pedaling cadence was kept constant during all the tests at a value identified during the familiarization session. For a graphical representation of the workload during the sinusoidal tests see Figure 1.

### Trapezoidal test

This test was designed to verify to what extent ventilatory and other physiological variables are associated with perceived exertion when the workload is self‐paced by the participant to match a predefined trapezoidal template of RPE. A computer screen was placed in front of the participant, and the RPE required by the test was displayed every second using a custom MATLAB program. Participants were asked to self‐pace the workload in order to have a perceived exertion value matching as closely as possible the RPE value displayed on the screen at each time point. To exclude any confounding effect of pedaling cadence variation on ventilatory responses, the ergometer was set in the isokinetic mode, and the pedaling cadence corresponded to that used during the sinusoidal tests. This ergometer setting allows the participant to adjust the workload promptly by varying the torque applied to the pedals.

The trapezoidal test lasted 41 min and consisted of three trapezoidal bouts of 11 min, each of which was preceded and followed by 2 min at a constant value of RPE (i.e., 11). Each trapezoidal bout consisted of a 3‐min linear increase in RPE from 11 to 18, 5 min at a constant RPE of 18 and a 3‐min linear decrease in RPE from 18 to 11. For a graphical representation of the trapezoidal test see Figure 4. The trapezoidal template of RPE used for this test was set up following pilot testing. The trapezoidal test was deliberately very demanding to observe a substantial decrease in workload from the first to the third bout.

Data from all the variables measured during the trapezoidal test were interpolated, extrapolated every second, and averaged over 60‐sec periods. The time course of the physiological and mechanical variables was compared between the first and the third trapezoidal bouts. The second trapezoidal bout was not included in the comparison because it was mainly introduced to increase the demand of the overall test and therefore to maximize the differences in workload between the first and the third bout. All the 41 data points were used for the correlations detailed in the “Statistical analysis” section.

### Cardiorespiratory variables

$\dot{V}O_{2}$, $\dot{V}{\text{CO}}_{2}$, ${\dot{V}}_{E}$, *f*_R_, *V*_T_, P_ETCO2_, P_ETO2_ and HR were measured breath‐by‐breath using a metabolic cart (Quark b2, Cosmed, Rome, Italy). Appropriate calibration procedures were performed following the manufacturer's instructions. Breath‐by‐breath data were filtered for errant breaths (i.e., values resulting from sighs, swallows, coughs, etc.) by deleting values greater than 3 standard deviations from the local mean.

### EMG data

Surface EMG was recorded in a single‐differential configuration from the left vastus lateralis (VL) muscle with a multichannel amplifier (3 dB bandwidth, band‐pass filter 10–500 Hz, gain = 200; EMG‐USB2+, OT Bioelettronica, Turin, Italy). The EMG signal was acquired with a 4‐electrode adhesive linear array (10 mm interelectrode distance, OT bioelettronica, Turin, Italy), sampled at 2048 Hz, and processed off‐line using a custom MATLAB (R2016a Mathworks, Natick, MA) program.

Before applying the electrodes, the skin was shaved, slightly abraded with abrasive paste (Meditec‐Every, Parma, Italy) and cleaned with ethanol. Subsequently, the adhesive array was placed along the estimated fiber orientation between the most distal location of the innervation zone and the distal tendon region. The position of the adhesive array was registered with dermatological ink in the first visit and it was replicated during subsequent visits.

Each pedal cycle was identified by means of a trigger signal obtained from a magnetic sensor placed top dead center on the carter of the ergometer. To identify the EMG burst for each pedal cycle, the raw EMG signal was rectified and filtered with a 5th order low‐pass 5‐Hz Butterworth filter to obtain a linear envelope. Subsequently, within each pedal cycle, the onset and offset points of the EMG burst were established as the points in which muscle activity exceeded 20% of the peak value of the envelope (Hug and Dorel 2009). For each burst identified, amplitude analysis was performed by computing the root mean square (RMS) of the EMG signal. Subsequently, RMS data were interpolated and extrapolated every second to allow for the calculation of the coefficients of the Fourier series described in the “Sinusoidal analysis” section.

### Mental demand and subjective rating of performance

After each sinusoidal or trapezoidal test, participants were asked to rate the mental demand subscale (how much mental and perceptual activity was required) and the performance subscale (how successful do you think you were in accomplishing the goals of the task set by the experimenter) of the National Aeronautics and Space Administration Task Load Index scale (NASA‐TLX) (Hart and Staveland 1988). For both subscales, participants were asked to provide a score on a visual analogic continuum from 0 to 20 between two poles, where for mental demand, 0 and 20 corresponded to low and high, respectively, while for performance, conversely, 0 and 20 corresponded to good and poor, respectively. Mental demand was quantified because ${\dot{V}}_{E}$ and *f*_R_ are influenced by cognitive tasks (Grassmann et al. 2016) even when performed during exercise (Acevedo et al. 2006). This is important in the light of the possibility that the modality of the RPE rating used during the sinusoidal tests and the inherent difficulty of the task performed during the trapezoidal test may have led the participants to perform a dual task exercise (i.e., a cognitive task performed during exercise). Performance was measured to provide quantitative information on the correct execution of the tasks.

### Sinusoidal analysis

For all the variables measured during the sinusoidal tests, data were linearly interpolated and extrapolated every second. In line with classical analysis procedures (Wigertz 1970; Casaburi et al. 1977), data from different sinusoidal cycles were time aligned and averaged to reduce the influence of random fluctuations. For all the four sinusoidal tests, the first 8 min were removed from the analysis to eliminate from the calculation the initial transient phase following the application of the sinusoidal workload. Therefore, 2 out of 3 cycles and 8 out of 12 cycles were averaged for the 8‐min period sinusoidal tests and the 2‐min period sinusoidal tests, respectively.

Amplitude (*A*) and phase lag (*φ*) were obtained by Fourier analysis from second‐by‐second average sinusoidal responses (Casaburi et al. 1977). Briefly, the *a* and *b* coefficients of the Fourier series were determined as follows:

$$a_{k}=\frac{2}{T}\sum_{t=0}^{T}\overset{¯}{x}\left(t\right)\cos\left(k\frac{2\pi }{T}t\right)\;\Delta t$$

$$b_{k}=\frac{2}{T}\sum_{t=0}^{T}\overset{¯}{x}\left(t\right)\sin\left(k\frac{2\pi }{T}t\right)\;\Delta t$$

where *k* is equal to the number of harmonics considered (*k *= 1 for the fundamental component), *T* is the period of the workload sinusoid (i.e., 120 sec for M_2 and M‐S_2; 480 sec for M_8 and M‐S_8), ▵*t* is the time interval between data points (1 sec), and $\overset{¯}{x}t$ is the averaged response for the time (*t*). The A and *φ* are calculated as follows:

$$A=\sqrt{b^{2}+a^{2}}$$

$$\varphi_{\mathrm{rad}}=\arctan\;\left(b/a\right)$$

To obtain an indication of the linearity of the response of the measured variables, the first three harmonics were computed. A and *φ* of the *k*th harmonic were obtained considering *k* alternatively equal to 1, 2, and 3. Specifically, the percentage contribution of the second and third harmonics to the fundamental component was considered for providing information on the linearity of the response (Wigertz 1970; Bakker et al. 1980). Note that we are interested in the accuracy of using the fundamental harmonic to describe the measured variables in terms of A and *φ*, with the aim of helping the physiological interpretation of our findings, but not to provide a rigorous mathematical identification of linear and non‐linear variables. For the same reason, A and *φ* were also computed for M‐S_2 and M‐S_8 to provide a simple characterization of the responses, despite the possibility that some of the measured variables may not show linear responses during moderate‐to‐severe fluctuations in workload. All the analyses reported in this section were performed in a MATLAB environment.

For each participant, second‐by‐second data of the average sinusoidal cycle were averaged into 20 segments of 6 s and 24 sec for the 2‐min sinusoidal tests and the 8‐min sinusoidal tests, respectively, in order to obtain 20 data points for each variable and sinusoidal test. Correlations were then computed as detailed in the following section.

### Statistical analysis

Statistical analyses were conducted using IBM SPSS Statistics 23 (SPSS Inc, Chicago, IL). A two‐way repeated‐measures ANOVA (period × intensity) was used to compare amplitude and phase lag across the four sinusoidal tests for physiological and perceptual variables, as well as to analyze mental demand and performance values from the NASA‐TLX scale. A two‐way repeated‐measures ANOVA (bout × time) was used to compare the time course of mechanical, physiological, and perceptual variables between the first and third trapezoidal bouts. When the sphericity assumption was violated, the Greenhouse–Geisser adjustment was performed. In case of a significant bout × time interaction, a paired Student's *t*‐test was used to test the simple main effect of bout at each time point. Partial eta squared (*ƞ*_P_^*2*^) effect sizes were calculated; an effect of *ƞ*_P_^2* *^≥ 0.01 indicates a small effect, *ƞ*_P_^2* *^≥ 0.059 a medium and *ƞ*_P_^2* *^ ≥ 0.138 a large effect (Cohen 1988).

Within‐subjects correlation coefficients (*r*) were computed for the correlations between relevant physiological and perceptual variables, using the method described by Bland and Altman (1995). This method adjusts for repeated observations within participants, using multiple regression with “participant” treated as a categorical factor using dummy variables. Specifically, the correlations between: $\dot{V}{\text{CO}}_{2}$ and ${\dot{V}}_{E}$, $\dot{V}{\text{CO}}_{2}$ and *V*_T_, and RMS and *f*_R_ were obtained during the trapezoidal test; the correlations between: RMS and *f*_R_, RPE and *f*_R_, $\dot{V}{\text{CO}}_{2}$ and ${\dot{V}}_{E}$, $\dot{V}{\text{CO}}_{2}$ and *V*_T_ were obtained during the sinusoidal tests. Pearson's correlation coefficients (*r*) were computed when correlating the A and *φ* of $\dot{V}{\text{CO}}_{2}$ with the A and *φ* of ${\dot{V}}_{E}$, *V*_T_, and *f*_R_.

A *P* < 0.05 was considered statistically significant in all analyses. The results are expressed as means (±SD) in the text and the Table, and as means (±SE) in the Figures.

## Results

The $\dot{V}O_{2\mathrm{peak}}$ and the PPO measured during the ramp incremental test were 4616 ± 406 mL·min^−1^ and 437 ± 42 W, respectively. The workload associated with VT1 was 198 ± 19 W. Consequently, the highest workload reached during the moderate‐intensity and the moderate‐to‐severe‐intensity sinusoidal tests was 178 ± 17 and 365 ± 34 W, respectively.

### Sinusoidal tests

**Figure 2:**
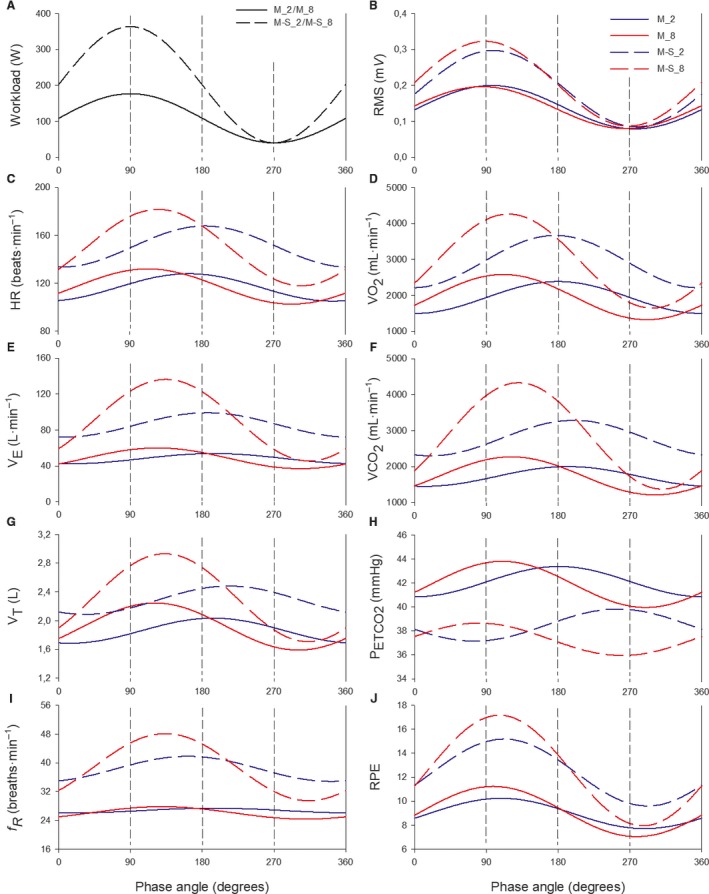
Group mean fitted sinusoidal responses for mechanical, physiological and perceptual variables as a function of the phase angle during the four sinusoidal tests. Workload is depicted in panel (A) for M_2 and M_8 (solid line) and for M‐S_2 and M‐S_8 (dashed line). For panels B–J, variables from the 2‐min and 8‐min sinusoidal tests are depicted in blue and red, respectively; the moderate tests and moderate‐to‐severe tests are depicted by solid and dashed lines, respectively.

Figure 1 shows the group's average response over time of mechanical, physiological and perceptual variables during the four sinusoidal tests. Of note is the fact that *f*_R_ is the only variable that shows no sinusoidal fluctuations during the M_2 test. For the four sinusoidal tests, the amplitude and phase lag of the physiological and perceptual variables are reported in Table 1, and the time course of these variables is depicted in Figure 2 as a function of the phase angle. For the phase lag (in degrees), an interaction (*P* < 0.001; *ƞ*_P_^2^ > 0.777) was observed for ${\dot{V}}_{E}$ and $\dot{V}O_{2}$ but not for the other variables reported in Table 1, while all the variables showed a main effect of period (*P* < 0.005; *ƞ*_P_^2^ > 0.548) except for *f*_R_. A main effect of intensity (*P* < 0.032; *ƞ*_P_^2^ > 0.357) was found for *V*_T_, $\dot{V}O_{2}$, $\dot{V}{\text{CO}}_{2}$, RPE and HR, but not for the other variables. While statistical analysis was only conducted on phase lag values in degrees, Table 1 also shows the phase lag in seconds to facilitate the physiological interpretation of our findings. For the amplitude, all the variables showed an interaction (*P* < 0.025; *ƞ*_P_^2^ > 0.384) except for P_ETCO2_, and a main effect of period (*P* < 0.007; *ƞ*_P_^2^ > 0.515) except for RMS. A main effect of intensity was observed for all the variables reported in Table 1 (*P* < 0.001; *ƞ*_P_^*2*^ > 0.815) except for P_ETCO2_. For all the variables shown in Figures 1, 2 and reported in Table 1, the number of participants included in the analysis is 12, except for HR (10 participants) because of technical problems that occurred in two tests.

**Table 1:** Amplitude and phase lag (in degrees and seconds) of physiological and perceptual variables for the four sinusoidal tests

	$\dot{V}O_{2}$	$\dot{V}{\text{CO}}_{2}$	P_ETCO2_	${\dot{V}}_{E}$	*V*_T_	*f*_R_	HR	RPE	RMS
	(mL·min^−1^)	(mL·min^−1^)	(mmHg)	(L·min^−1^)	(L)	(breaths·min^−1^)	(beats·min^−1^)		(m*V*)
A	a ^,^ b ^,^ c	a ^,^ b ^,^ c	a	a ^,^ b ^,^ c	a ^,^ b ^,^ c	a ^,^ b ^,^ c	a ^,^ b ^,^ c	a ^,^ b ^,^ c	b ^,^ c
M_2	446 ± 105	277 ± 76	1.3 ± 0.4	5.8 ± 2.0	0.18 ± 0.04	1.0 ± 0.3	11.5 ± 3.5	1.3 ± 0.8	0.06 ± 0.02
M_8	629 ± 84	529 ± 66	1.9 ± 0.7	11.6 ± 1.8	0.33 ± 0.12	2.0 ± 1.5	14.7 ± 3.6	2.1 ± 0.5	0.06 ± 0.01
M‐S_2	734 ± 147	502 ± 113	1.6 ± 0.7	13.9 ± 4.9	0.20 ± 0.05	3.7 ± 2.1	17.2 ± 3.1	3.0 ± 1.5	0.11 ± 0.03
M‐S_8	1309 ± 170	1488 ± 174	2.0 ± 0.8	45.8 ± 11.5	0.62 ± 0.18	9.7 ± 4.3	31.9 ± 4.4	4.7 ± 0.9	0.12 ± 0.04
*φ* (degree)	a ^,^ b ^,^ c	a ^,^ b	a	a ^,^ c	a ^,^ b		a ^,^ b	a ^,^ b	a
M_2	−91 ± 6	−103 ± 8	−94 ± 20	−106 ± 12	−104 ± 18	−49 ± 105	−74 ± 5	−18 ± 14	−7 ± 6
M_8	−22 ± 3	−32 ± 4	−20 ± 5	−34 ± 6	−32 ± 10	−56 ± 39	−23 ± 4	−8 ± 11	5 ± 3
M‐S_2	−88 ± 7	−112 ± 12	−42 ± 154	−101 ± 15	−125 ± 16	−79 ± 34	−95 ± 7	−31 ± 23	−8 ± 6
M‐S_8	−28 ± 4	−41 ± 6	24 ± 54	−46 ± 9	−44 ± 11	−46 ± 16	−35 ± 4	−18 ± 14	2 ± 6
*φ* (s)									
M_2	30 ± 2	35 ± 3	31 ± 7	35 ± 4	35 ± 6	16 ± 35	25 ± 2	6± 5	3 ± 2
M_8	29 ± 4	42 ± 5	27 ± 7	46 ± 7	43 ± 14	75 ±52	31 ± 6	10 ± 15	−6 ± 4
M‐S_2	29 ± 2	38 ± 4	14 ± 5	34 ± 5	42 ± 6	26 ± 11	32 ± 2	10 ± 8	3 ± 2
M‐S_8	38 ± 5	55 ± 8	−30 ± 73	60 ± 12	58 ± 14	61 ± 21	47 ± 5	24 ± 19	−3 ± 8

$\dot{V}O_{2}$, oxygen uptake; $\dot{V}{\text{CO}}_{2}$, carbon dioxide output; P_ETCO2_, end‐tidal partial pressure of carbon dioxide; ${\dot{V}}_{E}$, minute ventilation; *V*_T_, tidal volume; *f*_R_, respiratory frequency; HR, heart rate; RPE, rating of perceived exertion; RMS, root mean square; *A*, amplitude; *φ*, phase lag; M_2, moderate‐intensity test with sinusoidal period of 2 min; M_8, moderate‐intensity test with sinusoidal period of 8 min; M‐S_2, moderate‐to‐severe‐intensity test with sinusoidal period of 2 min; M‐S_8, moderate‐to‐severe‐intensity test with sinusoidal period of 8 min. Values are means ± SD. Statistical analysis was not performed on phase lag values in seconds, which are only provided to facilitate the physiological interpretation of the data.

a Main effect of period (*P* < 0.05)

b Main effect of intensity (*P* < 0.05).

c Significant interaction (*P* < 0.05).

**Figure 3:**
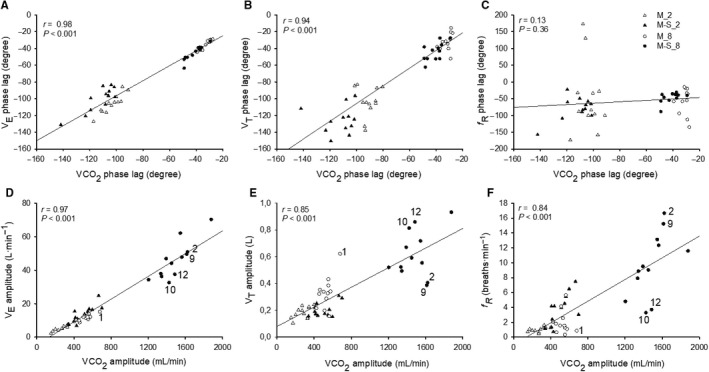
Correlations between the phase lag of $\dot{V}{\text{CO}}_{2}$ and the phase lag of ${\dot{V}}_{E}$ (A), *V*_T_ (B) and *f*_R_ (C) for M_2 (open triangles), M_8 (open circles), M‐S_2 (filled triangles) and M‐S_8 (filled circles). Correlations between the amplitude of $\dot{V}{\text{CO}}_{2}$ and the amplitude of ${\dot{V}}_{E}$ (D), *V*_T_ (E) and *f*_R_ (F) for M_2 (open triangles), M_8 (open circles), M‐S_2 (filled triangles) and M‐S_8 (filled circles). For the correlations shown in panels D, E and F, the numbers within each panel depict different participants. These numbers help to highlight that when the amplitude of *f*_R_ is relatively low (1, 10, and 12), *V*_T_ is relatively high; when the amplitude of *f*_R_ is relatively high (2 and 9), *V*_T_ is relatively low. The reciprocal changes between *V*_T_ and *f*_R_ determine a stronger correlation between the amplitude of $\dot{V}{\text{CO}}_{2}$ and that of ${\dot{V}}_{E}$ compared to the correlations between the amplitude of $\dot{V}{\text{CO}}_{2}$ and those of *V*_T_ and *f*_R_.

When considering the four sinusoidal tests together, the Bland–Altman correlation analysis showed a larger correlation between *f*_R_ and RPE (*P* < 0.001; *r* = 0.74) compared to the correlation between *f*_R_ and RMS (*P* < 0.001; *r* = 0.53). A large correlation was observed between *V*_T_ and $\dot{V}{\text{CO}}_{2}$ (*P* < 0.001; *r* = 0.91) and between ${\dot{V}}_{E}$ and $\dot{V}{\text{CO}}_{2}$ (*P* < 0.001; *r* = 0.98). Figure 3 shows the correlations (with *r* and *P* values) of the A and *φ* of $\dot{V}{\text{CO}}_{2}$ with the A and *φ* of ${\dot{V}}_{E}$, *V*_T_, and *f*_R_. The correlations between $\dot{V}{\text{CO}}_{2}$ and ${\dot{V}}_{E}$ were higher than the correlations between $\dot{V}{\text{CO}}_{2}$ and both *f*_R_ and *V*_T_. Furthermore, Figure 3 highlights some individual values in order to point out that the close correlation observed between ${\dot{V}}_{E}$ and $\dot{V}{\text{CO}}_{2}$ is guaranteed by the reciprocal changes between *f*_R_ and *V*_T_. Indeed, when the amplitude of *f*_R_ is relatively low, *V*_T_ amplitude is relatively high; when the amplitude of *f*_R_ is relatively high, *V*_T_ amplitude is relatively low.

For M_2, harmonic analysis showed that the *f*_R_ amplitude of the second and third harmonic was 62 ± 34% and 42 ± 23% of that of the fundamental harmonic, thus indicating a nonlinear response of *f*_R_. Conversely, the contribution of the second and third harmonics was considerably lower for all the other variables measured, with *V*_T_ showing values of 23 ± 11% and 12 ± 8% for the second and third harmonics, respectively. Compared to the M_2 test, *f*_R_ was better described by the fundamental harmonic in the other sinusoidal tests, with the contribution of the second and third harmonics being 35 ± 36%, 31 ± 36%, 16 ± 13% and 40 ± 39%, 24 ± 24%, 11 ± 8% for M_8, M‐S_2 and M‐S_8, respectively. The contributions of the second and third harmonics for all the other variables were generally lower than those observed for *f*_R_, with *V*_T_ values being 10 ± 11%, 21 ± 15%, 11 ± 3% and 11 ± 8%, 16 ± 11%, 6 ± 4% for M_8, M‐S_2 and M‐S_8, respectively.

Participants reported a relatively high mental demand after the four sinusoidal tests (M_2 = 10.8 ± 5.3; M_8 = 10.5 ± 5.3; M‐S_2 = 12.7 ± 3.5; M‐S_8 = 13.9 ± 3.3), with a main effect of intensity (*P* < 0.004; *ƞ*_P_^*2*^ > 0.557), but no significant main effect of period or interaction. Participants reported being generally successful in accomplishing the task set during the sinusoidal tests (M_2 = 4.8 ± 2.4; M_8 = 5.0 ± 3.3; M‐S_2 = 4.7 ± 1.8; M‐S_8 = 4.9 ± 3.0), with no significant main effects of period and intensity or interaction.

### Trapezoidal test

**Figure 4:**
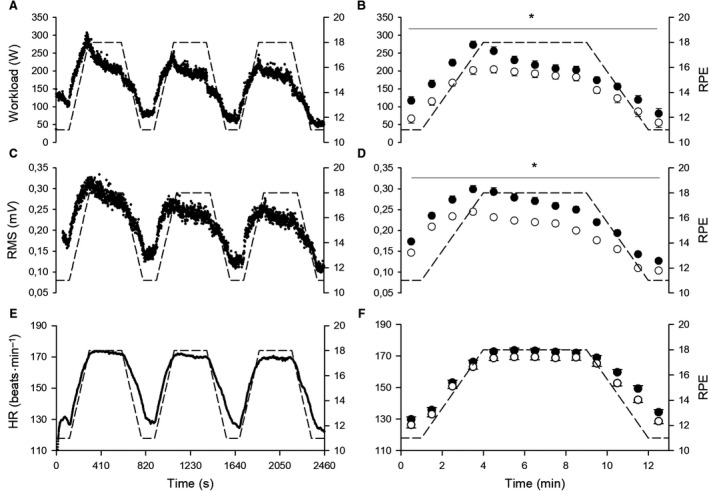
In the left panels, group mean response of second‐by‐second data for workload (A), RMS (C) and HR (E) during the entire trapezoidal test. The RPE required by the test is depicted in dashed lines. In the right panels, 60‐sec average values of workload (B), RMS (D) and HR (F) for the first (filled circles) and the third (open circles) trapezoidal bouts. When a significant bout × time interaction was found, * depicts significant simple main effect of bout (*P* < 0.05).

**Figure 5:**
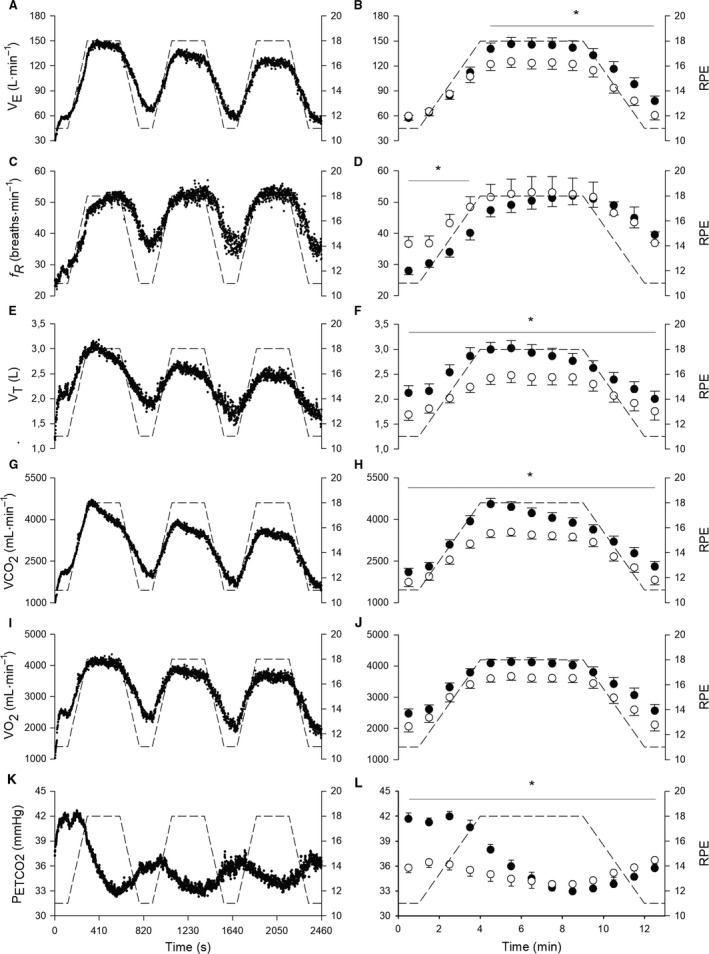
In the left panels, group mean response of second‐by‐second data for ${\dot{V}}_{E}$ (A), *f*_R_ (C), *V*_T_ (E), $\dot{V}{\text{CO}}_{2}$ (G)_,_ $\dot{V}O_{2}$ (I) and P_ETCO__2_ (K) during the entire trapezoidal test. The RPE required by the test is depicted in dashed lines. In the right panels, 60‐sec average values of ${\dot{V}}_{E}$ (B), *f*_R_ (D), *V*_T_ (F), $\dot{V}{\text{CO}}_{2}$ (H), $\dot{V}O_{2}$ (J) and P_ETCO__2_ (L) for the first (filled circles) and the third (open circles) trapezoidal bouts. When a significant bout × time interaction was found, * depicts significant simple main effect of bout (*P* < 0.05).

Figures 4 and 5 depict the second‐by‐second time course of mechanical and physiological responses during the entire trapezoidal test in the left panels, and the comparison between 60‐s average values of the first and third trapezoidal bouts in the right panels. The number of participants included in the analysis is 12 for all the variables, except for HR (10 participants) and RMS (11 participants) because of technical problems that occurred with two participants in the first case and 1 participant in the second case.

All the variables showed a main effect of time and bout (*P* < 0.040; *ƞ*_P_^*2*^ > 0.391), except for *f*_R_ that did not show a main effect of bout. An interaction was observed for all the variables (*P* < 0.004; *ƞ*_P_^2^ > 0.211), except for $\dot{V}O_{2}$ and HR. When an interaction was found, Figures 4 and 5 show where a simple main effect of bout was observed. Unlike any other variable, *f*_R_ did not show any significant difference when comparing the first and third bout during the 5 min at the fixed RPE value of 18.

A large correlation was observed between *V*_T_ and $\dot{V}{\text{CO}}_{2}$ (*P* < 0.001; *r* = 0.91) and between ${\dot{V}}_{E}$ and $\dot{V}{\text{CO}}_{2}$ (*P* < 0.001; *r* = 0.94), while a small correlation was observed between *f*_R_ and RMS (*P* < 0.002; *r* = 0.17). Participants reported that the trapezoidal test was mentally demanding (16.5 ± 2.6), but their ability to perform the task was generally rated as good (5.1 ± 2.8).

## Discussion

The present study proposes a series of exercise manipulations collectively aiming at furthering our understanding of the overlooked differential control of *f*_R_ and *V*_T_ during exercise. The main findings of the present study are as follows: (1) *f*_R_ changes more with RPE than with workload, metabolic variables, *V*_T_ or the amount of muscle activation, but it dissociates from RPE during moderate exercise; (2) Unlike *f*_R_, *V*_T_ mediates the close association observed between ${\dot{V}}_{E}$ and $\dot{V}{\text{CO}}_{2}$ in any exercise condition, while continuously adjusting its value on the basis of *f*_R_ levels. Collectively, our findings provide a novel framework for understanding how *f*_R_ and *V*_T_ are regulated during exercise, with important implications for the interpretation of previous findings and for the design of future research. For instance, our findings suggest a novel interpretation of the long‐debated issue of ventilatory control during sinusoidal exercise. The emerging conceptual framework clarifies the importance of differentiating between *f*_R_ and *V*_T_ to further our understanding of exercise hyperpnoea.

### Control of *f*_R_

The present findings collectively reveal that *f*_R_ has a very peculiar response to exercise, which is more influenced by RPE levels than by workload levels, the amount of muscle activation or by metabolic requirements. When changes in workload determine the attainment of relatively low RPE values (approximately below 11 of RPE), *f*_R_ shows either no change at all or only a mild response, as we observed during the moderate‐intensity sinusoidal tests. Conversely, when changes in workload determine more substantial variations in RPE (well above 11), *f*_R_ also shows a substantial response. This was observed during the moderate‐to‐severe sinusoidal tests and the trapezoidal test, and is in line with the close association found between RPE and *f*_R_ during different high‐intensity protocols (Robertson and Noble 1997; Nicolò et al., 2014, 2016, 2017b). Therefore, *f*_R_ is sensitive to exercise conditions where relatively high, but not low, levels of RPE are reached.

This feature of *f*_R_ is particularly evident when comparing the four sinusoidal tests. When low values of RPE were reached, *f*_R_ showed almost no change, especially in the test with the smallest RPE amplitude (M_2 test), where a nonlinear response of *f*_R_ was observed. Conversely, when more pronounced variations in RPE were observed (M‐S_2 and M‐S_8), *f*_R_ showed evident sinusoidal fluctuations, which were larger in the test with the greatest amplitude in RPE (M‐S_8), despite the same workload amplitude of M‐S_2. Furthermore, considering the four sinusoidal tests together, *f*_R_ showed a stronger association with RPE than with the amount of muscle activation; indeed, the correlation between *f*_R_ and RPE (*r* = 0.74) was higher than that between *f*_R_ and RMS (*r* = 0.53).

The mild response of *f*_R_ to moderate‐intensity sinusoidal exercise is important because it reveals that *f*_R_ does not mediate the close association between ${\dot{V}}_{E}$ and $\dot{V}{\text{CO}}_{2}$ found in the present study and a number of previous studies (Casaburi et al. 1977, 1995; Haouzi 2006; Bakker et al. 1980; Miyamoto et al. 1983; Fukuoka et al. 2017). The observed response of *f*_R_ is in line with the few reports on the responses of *f*_R_ and *V*_T_ during sinusoidal exercise (Bakker et al. 1980; Miyamoto et al. 1983). However, no attempt had previously been made to interpret these remarkable findings. We argue that the response of *f*_R_ is evidence against *f*_R_ being regulated by metabolic stimuli, and evidence of the fact that the main inputs regulating *f*_R_ have a relatively low magnitude during moderate exercise. As discussed below, these findings lead to a novel interpretation of the long‐debated issue of ventilatory control during sinusoidal exercise (Haouzi, 2006, 2015; Forster et al. 2012).

From the trapezoidal test, it is further evident that *f*_R_ is more associated with perceived exertion than with workload, or with the amount of muscle activation or with metabolic markers. Indeed, the workload decreased from the first to the third trapezoidal bout as a result of the time spent at high levels of RPE, and a similar decrease in RMS, $\dot{V}O_{2}$, $\dot{V}{\text{CO}}_{2}$ and *V*_T_ was observed. Conversely, *f*_R_ did not show any decrease from the first to the third trapezoidal bout, and it resembled the plateau of RPE during the 5 min at 18 of RPE, unlike any other variable measured in the present study. However, *f*_R_ and RPE were partially dissociated at lower RPE values (see Discussion below).

The dissociation observed between *f*_R_ and metabolic variables provides evidence in support of the proposition that *f*_R_ is not substantially regulated by metabolic stimuli (Nicolò et al. 2017a). This notion seems to hold true during both exercise (Nicolò et al. 2017b) and non‐exercise conditions (Tipton et al. 2017; Nicolò et al. 2017a). Even when *f*_R_ appears to respond to metabolic stimuli like hypercapnia, it turns out that the *f*_R_ response may be mediated by the increase in *V*_T_ (volume feedback) and/or by hypercapnia‐induced sensations rather than by a direct effect of hypercapnia on *f*_R_ (Nicolò et al. 2017a). This has been convincingly shown in both animals (Borison et al. 1977) and humans (Guz et al. 1966). Nevertheless, we cannot exclude that some of the metabolic stimuli may have contributed to regulating *f*_R_ in the present study because the need to reproduce “real” exercise conditions limited the possibility of measuring some metabolic markers such as pH, potassium, bicarbonate and arterial partial pressures of O_2_ and CO_2_. However, there is evidence that some of these stimuli may not drive *f*_R_ during exercise (Clark et al. 1980; Busse et al. 1991; Forster et al. 2012). Furthermore, several studies show a dissociation between *f*_R_ and blood lactate in a number of experimental conditions (Busse et al. 1991; Hayashi et al. 2006; Forster et al. 2012; Nicolò et al. 2017c). Therefore, while the observed response of *f*_R_ may somehow resemble the response of blood lactate to different exercise‐intensity domains, there is clear evidence that blood lactate does not drive *f*_R_.

While there is substantial evidence suggesting that *f*_R_ is not substantially regulated by metabolic stimuli, it is more difficult to identify the inputs driving *f*_R_ during exercise, and their relative contributions. Nevertheless, the present findings support the notion that the magnitude of central command (i.e., the activity of motor and premotor areas of the brain relating to voluntary muscle contractions) contributes to regulating *f*_R_ during exercise (Nicolò et al. 2017b). Central command is an important regulator of both *f*_R_ and perceived exertion (Thornton et al. 2001; Green et al. 2007; de Morree et al., 2012, 2014; Zénon et al. 2015; Nicolò et al. 2017b), and this may explain why *f*_R_ and RPE are closely associated during a variety of exercise conditions (Robertson and Noble 1997; Nicolò et al., 2014, 2016, 2017b), including some of those used in the present study. Accordingly, a relevant finding of this study is that *f*_R_ is more associated with RPE than with the amount of muscle activation. This is interesting in the light of electroencephalographic data showing that the magnitude of central command and RPE can change independently of workload and the amount of muscle activation (de Morree et al. 2014). Likewise, in cardiovascular physiology, central command is defined as an effort‐induced modulation of autonomic function which can be independent of force production (Williamson et al. 2006). Moreover, neuroimaging data show that actual muscle contraction is not essential for inducing a central‐command‐mediated increase in *f*_R_ (Thornton et al. 2001; Green et al. 2007). Therefore, the activity of brain areas relating to voluntary muscle contraction – rather than the amount of muscle activation *per se* – may contribute to regulating *f*_R_.

The partial dissociation observed between *f*_R_ and the amount of muscle activation during the sinusoidal and trapezoidal tests may also suggest that muscle afferent feedback (at least mechanical afferent feedback) is not the primary driver of *f*_R_ during high‐intensity exercise. This interpretation is in line with experimental evidence showing that the proportional contribution of muscle afferent feedback to *f*_R_ regulation decreases with the increase in exercise intensity (Amann et al. 2010). Furthermore, during high‐intensity exercise, *f*_R_ can increase as a consequence of the increase in the magnitude of central command with no change (Marcora et al. 2008) or with a decrease (Amann et al. 2008) in muscle afferent feedback.

In contrast to our hypothesis, we found a partial dissociation between the time courses of RPE and *f*_R_ during sinusoidal and trapezoidal tests. Indeed, the phase lag of *f*_R_ was substantially longer than that of RPE in the sinusoidal tests, and the trapezoidal variations in *f*_R_ were delayed compared to the variations in RPE required by the test. However, these findings do not necessarily suggest that, in any exercise condition, the response of *f*_R_ is delayed compared to the sensation of perceived exertion. Indeed, it has been repeatedly observed that *f*_R_ shows a rapid response to abrupt changes in workload during intermittent exercise (Nicolò et al., 2017b, 2017c). Some factors may have influenced the observed *f*_R_ response. For instance, it is of note that the present study is unique in the attempt to describe in detail the moment‐by‐moment changes in RPE. In fact, the cognitive demand imposed on the participants to obtain such temporal precision in the measure of RPE resulted in the participants performing a dual task exercise, that is a cognitive task performed during exercise. This is confirmed by the relatively high values of mental demand reported after the sinusoidal tests, including the tests performed at moderate intensity. Indeed, during the sinusoidal tests, participants were in a constant state of alertness because they were asked to detect potential changes in RPE continuously, while at the same time keeping the pedaling cadence as close as possible to the target cadence. Similarly, the rated mental demand after the trapezoidal test was high because of the inherent difficulty of the task, especially during the variable‐RPE portions.

Recognizing that the participants were under a dual‐task condition is important because *f*_R_ is very sensitive to cognitive tasks performed not only at rest (Grassmann et al. 2016), but also during exercise, even in trained individuals (Acevedo et al. 2006). Moreover, it has been found that a cognitive task performed during exercise may increase *f*_R_ without affecting RPE (Acevedo et al. 2006). This suggests that central neural inputs other than central command may have contributed to regulating *f*_R_ in the present study, like the so called “wakefulness drive to breathe”, that is, an increase in central neural activity or arousal, similar to alertness or awareness (Bell and Duffin 2004).

It cannot be excluded that the partial dissociation found between the time courses of RPE and *f*_R_ may have been influenced in some instances by errors committed when rating RPE. For instance, it may have been difficult to differentiate perceived exertion from the sensation of force (Luu et al. 2011) during the moderate‐intensity sinusoidal tests, because RPE was low and the torque varied in a sinusoidal fashion, the pedaling cadence being constant. However, careful attention was devoted to familiarizing the participants with the RPE scale and related procedures and tasks (trapezoidal test), and well‐trained participants were recruited to guarantee the correct execution of the tests. We also used a rating scale to quantitatively assess the ability of the participants to provide RPE values frequently (sinusoidal tests) and to perform the task (trapezoidal test), and good values were generally reported. Therefore, these data suggest that the tasks required of the participants were feasible and well performed, although cognitively demanding.

The response of *f*_R_ may have been affected by other factors along with those aforementioned. Exercise‐induced variations in body temperature affect *f*_R_, although the underlying mechanisms are not well understood (Hayashi et al. 2006). Accordingly, the increase in *f*_R_ over time observed during the sinusoidal tests (especially in the M‐S tests) may have been influenced by an increase in body temperature. However, sinusoidal changes in oesophageal and muscular temperature show a substantially longer phase lag (Todd et al. 2014) compared to the phase lag observed for *f*_R_ in the present study, making it difficult to attribute fluctuations in *f*_R_ during sinusoidal exercise to those of body temperature. It is more plausible that some properties of the respiratory neurons or phenomena observed in the brain after exercise cessation can partly explain the time course differences observed between *f*_R_ and RPE during sinusoidal and trapezoidal tests. The so‐called “short‐term potentiation” phenomenon has been advocated to explain the latency shown by *f*_R_ after the removal of a given stimulus (Fregosi 1991), including exercise (Mateika and Duffin 1992). This hypothesis is based on the “potentiation” property of neurons (including respiratory neurons), that is, a stimulus‐like response with an exponential decay at the removal of the stimulus (Whipp and Ward 1998; Forster et al. 2012). More interestingly, neuroimaging evidence indicates that some of the cortical and subcortical areas involved in the control of movement and ventilation maintain their activity even after exercise termination, despite the absence of any activity from locomotor muscles (Fink et al. 1995). A similar phenomenon may occur during non‐abrupt fluctuations in workload, such as those proposed in the present study. In addition, given the link between *f*_R_ and emotions (Homma and Masaoka 2008), it cannot be excluded that the *f*_R_ response may have been influenced to some extent by changes in affective valence induced by exercise and/or by the cognitive tasks performed during exercise.

### Control of *V*_T_

A very different response compared to that of *f*_R_ was found for *V*_T_ in all the exercise tests. The phase of *V*_T_ was very similar to that of $\dot{V}{\text{CO}}_{2}$ during the sinusoidal tests and a strong correlation was found between *V*_T_ and $\dot{V}{\text{CO}}_{2}$ during all the tests, with the two variables showing a very similar time course. Therefore, *V*_T_ is closely linked with $\dot{V}{\text{CO}}_{2}$ irrespective of exercise intensity, sinusoidal periods, and perceived exertion levels. The fact that *V*_T_ mediates the association between ${\dot{V}}_{E}$ and $\dot{V}{\text{CO}}_{2}$ is a neglected observation, despite a large amount of data suggesting the existence of a close association between ${\dot{V}}_{E}$ and $\dot{V}{\text{CO}}_{2}$ (Casaburi et al. 1977, 1995; Whipp and Ward 1998; Haouzi, 2006, 2015; Forster et al. 2012; Fukuoka et al. 2017). This is especially true for research on sinusoidal exercise, where only a few studies have reported the responses of *f*_R_ and *V*_T_ without further discussing their different behavior (Bakker et al. 1980; Miyamoto et al. 1983).

However, the association between ${\dot{V}}_{E}$ and $\dot{V}{\text{CO}}_{2}$ turned out to be stronger than the association between *V*_T_ and $\dot{V}{\text{CO}}_{2}$ during sinusoidal and trapezoidal tests. Furthermore, the correlations between the amplitude of the ventilatory variables and that of $\dot{V}{\text{CO}}_{2}$ show reciprocal changes between *f*_R_ and *V*_T_ in order to match ${\dot{V}}_{E}$ with $\dot{V}{\text{CO}}_{2}$. For instance, when *f*_R_ amplitude is relatively high, *V*_T_ amplitude is relatively low; when *f*_R_ amplitude is relatively low, *V*_T_ amplitude is relatively high (Fig. 3). These findings are of note because if we assume that *f*_R_ does not respond directly to metabolic stimuli, it follows that *V*_T_ mediates the close association between ${\dot{V}}_{E}$ and $\dot{V}{\text{CO}}_{2}$ by adjusting its value on the level of *f*_R_, along with the magnitude of metabolic stimuli. Therefore, beyond the existence of a differential control of *V*_T_ and *f*_R_, it emerges that there is an unbalanced interdependence between *V*_T_ and *f*_R_. While *f*_R_ seems not to be substantially influenced by the levels of *V*_T_, at least until critical *V*_T_ levels are reached (Duffin et al. 2000; Sheel and Romer 2012), *V*_T_ appears to be constantly influenced by *f*_R_ in order to guarantee that ${\dot{V}}_{E}$ is matched to $\dot{V}{\text{CO}}_{2}$, irrespective of the specific value of *f*_R_. Accordingly, it is interesting to observe from Figures 1 and 5 and previous reports (Pearce and Milhorn 1977) that the second‐by‐second/breath‐by‐breath variability of ${\dot{V}}_{E}$ is somewhat smaller than the variability of *f*_R_ and *V*_T_, which therefore change reciprocally to reduce the variability in ${\dot{V}}_{E}$. However, our findings seem to suggest that *V*_T_ continuously adjusts on the basis of *f*_R_ levels, but not vice versa. This interpretation agrees with previous findings (Haouzi and Bell 2009; Ohashi et al. 2013), albeit mostly obtained during non‐exercise conditions. When *f*_R_ is voluntarily controlled, *V*_T_ adjusts on the basis of the levels of *f*_R_ and CO_2_ to keep alveolar ventilation constant irrespective of the experimental conditions tested, that is, increased dead space, hypercapnia, and light exercise (Haouzi and Bell 2009). Expanding on these results, Ohashi et al. (2013) found that at rest the correspondence between ${\dot{V}}_{E}$ and $\dot{V}{\text{CO}}_{2}$ is lost when voluntarily controlling *V*_T_ rather than *f*_R_, suggesting that *f*_R_ has no active role in guaranteeing the matching between ${\dot{V}}_{E}$ and $\dot{V}{\text{CO}}_{2}$.

The existence of an unbalanced interdependence between *V*_T_ and *f*_R_ is in line with the proposition that *f*_R_ and *V*_T_ fulfil different roles with different timings (Nicolò et al. 2017a). In a number of conditions where a fast increase in ventilation is induced by non‐metabolic stressors, *f*_R_ is rapidly driven by fast inputs while *V*_T_ fine‐tunes ventilation to account for the slow changes in metabolism (Tipton et al. 2017; Nicolò et al. 2017a). This happens when ${\dot{V}}_{E}$ increases rapidly in response to abrupt changes in workload during exercise (Nicolò et al. 2017b) or with the rapid onset of stressors like acute pain, panic, and cold (Tipton et al. 2017). Conversely, when metabolic stimuli are predominant and the magnitude of fast inputs is relatively low, *f*_R_ is almost unchanged and the increase in ${\dot{V}}_{E}$ is mediated by *V*_T_. This holds true during moderate hypercapnia, intermittent hypoxia, intravenous infusion of stress hormones and during a shivering‐induced increase in metabolic demand (Tipton et al. 2017; Nicolò et al. 2017a).

### A novel interpretation of ventilatory control during sinusoidal exercise

Data from sinusoidal exercise have long contributed to exacerbating the debate between the proponents of “neurogenic” or “metabolic” explanations for the mechanisms underlying exercise hyperpnoea (Whipp and Ward 1998; Haouzi, 2006, 2015; Forster et al. 2012). Indeed, findings from moderate‐intensity sinusoidal studies have led to the conclusion that ventilation follows metabolic stimuli and “neglects” other inputs like central command and afferent feedback (Haouzi, 2006, 2015; Forster et al. 2012), with the possible exception of muscle afferent feedback sensing vascular distension (Haouzi 2006). However, in the light of the differential control of *V*_T_ and *f*_R_, partly different conclusions can be drawn.

Findings from M_8 and especially M_2 suggest that the magnitude of the inputs regulating *f*_R_ does not change enough to produce a substantial change in *f*_R_ when moderate‐intensity sinusoidal variations are imposed. Conversely, with moderate‐to‐severe sinusoidal variations in workload, the magnitude of the inputs regulating *f*_R_ changes substantially, determining sinusoidal fluctuations in *f*_R_. Although the partial dissociation between *f*_R_ and RPE warrants caution, these findings may suggest that the magnitude of central command does not change substantially during moderate‐intensity sinusoidal variations in workload, while it does during moderate‐to‐severe variations (more in M‐S_8 than in M‐S_2 despite the same workload amplitude). This interpretation may help shed some light on the long‐debated apparent conflict between data from moderate‐intensity sinusoidal exercise and evidence supporting the contribution of central command to exercise hyperpnoea (Forster et al. 2012). Note that this proposition implies that central command is viewed in an effort perspective (Thornton et al. 2001; Williamson et al. 2006; Green et al. 2007; de Morree et al., 2012, 2014; Zénon et al. 2015; Nicolò et al. 2017b) rather than being merely represented by workload. Conversely, in respiratory physiology, workload is commonly viewed as an indicator of central command (Whipp and Ward 1998; Haouzi, 2006, 2015; Forster et al. 2012). On the other hand, *V*_T_ mediates the association between ${\dot{V}}_{E}$ and $\dot{V}{\text{CO}}_{2}$ either when *f*_R_ changes substantially (M‐S_2 and M‐S_8; high‐magnitude of fast inputs) or not (M_2 and M_8; low‐magnitude of fast inputs). Therefore, metabolic and non‐metabolic inputs may regulate ventilation by acting differently on *V*_T_ and *f*_R_. However, *V*_T_ and *f*_R_ do not merely reflect the magnitude of metabolic or non‐metabolic inputs because of the existence of an unbalanced interdependence between *f*_R_ and *V*_T_, which guarantees the coupling between ${\dot{V}}_{E}$ and $\dot{V}{\text{CO}}_{2}$. The suggested model of ventilatory control may account for exercise hyperpnoea in a variety of conditions, that is, during any exercise‐intensity domain and both steady state and non‐steady‐state conditions.

The present findings and interpretations have important implications for future research on the mechanisms regulating exercise hyperpnoea. A major conundrum in respiratory physiology is the identification of the mechanisms that link alveolar ventilation to CO_2_ exchange to guarantee that CO_2_/H^+^ homeostasis is maintained during moderate exercise (Forster et al. 2012). In view of the proposition that functional studies in “intact” humans should dictate the direction taken by the most fundamental research (Haouzi 2006), our findings suggest that this conundrum should be addressed by specifically looking at the control of *V*_T_ and at the mechanisms underlying the unbalanced interdependence between *f*_R_ and *V*_T_.

## Conclusion

By proposing a series of exercise manipulations, the present study provides a novel framework to further our understanding of the control of *f*_R_ and *V*_T_ during exercise. We observed a differential control of *f*_R_ and *V*_T_ across different exercise‐intensity domains, sinusoidal exercise periods, and perceived exertion levels. More specifically, *f*_R_ changes more with RPE than with workload, *V*_T_, metabolic variables or with the amount of muscle activation. However, *f*_R_ dissociates from RPE during moderate exercise. Unlike *f*_R_, *V*_T_ mediates the association between ${\dot{V}}_{E}$ and $\dot{V}{\text{CO}}_{2}$ by adjusting its value on the basis of *f*_R_ levels, hence suggesting the existence of an unbalanced interdependence between *f*_R_ and *V*_T_. These findings provide further insight into the importance of differentiating between *f*_R_ and *V*_T_ to improve our understanding of exercise hyperpnoea.

## Conflict of Interest

None declared.
